# Comparison of the safety, effectiveness, and usability of swab robot vs. manual nasopharyngeal specimen collection

**DOI:** 10.1016/j.heliyon.2023.e20757

**Published:** 2023-10-13

**Authors:** Jiun-Hao Yu, Sung-huai Hsieh, Chieh‐Hsiao Chen, Wen-Kuan Huang

**Affiliations:** a.Department of Emergency Medicine, China Medical University Hsinchu Hospital, China Medical University, Hsinchu, 30272, Taiwan; b.Graduate Institute of Management, Chang Gung University, Taoyuan City, Taiwan; c.Department of Information Technology System, China Medical University Hsinchu Hospital, Hsinchu, 30272, Taiwan; d.Department of Digital Health Innovation Master's Program, China Medical University, Taichung, 40402, Taiwan; e.China Medical University and Beigang Hospital, Yunlin County, Taiwan; f.Brain Navi Biotechnology Co., Ltd., Hsinchu County, 30261, Taiwan; g.School of Medicine, College of Medicine, Chang Gung University, Taoyuan City, Taiwan; h.Division of Hematology/Oncology, Department of Internal Medicine, Chang Gung Memorial Hospital at Linkou, Taoyuan, Taiwan

**Keywords:** Swab robot, Nasopharyngeal sampling, Respiratory tract infection, COVID-19 pandemic, SARS-CoV-2 testing, Specimen collection

## Abstract

**Background:**

Healthcare workers face a risk of infection during aerosol-generating procedures, such as nasal swabbing. Robot-assisted nasopharyngeal sampling aims to minimize this risk and reduce stress for healthcare providers. However, its effectiveness and safety require validation.

**Methods:**

We conducted a controlled trial with 80 subjects at two teaching hospitals and compared robot-collected vs manually-collected nasopharyngeal swabs. The primary outcomes included specimen quality and success rate of nasopharyngeal swab collection. We also recorded the pain index, duration of the collection, and psychological stress using a post-collection questionnaire.

**Results:**

During the study period, from September 23 to October 27, 2020, 40 subjects were enrolled in both the robotic and manual groups. The cycle threshold (Ct) value for nasopharyngeal specimens was statistically higher in the robotic group compared to the manual group (30.9 vs 28.0, p < 0.01). Both groups had Ct values under 35, indicating good quality specimens. In the robotic group, 3 out of 40 subjects required a second attempt at specimen collection, resulting in a success rate of 92.5 %. Further, although the pain levels were lower in the robotic group, the difference was not statistically significant (2.8 vs 3.6, p = 0.07). The manual group had a shorter sampling time, which was 29 s (201 vs 29, p < 0.05). However, when factoring in the time needed to put on personal protective equipment, the average time for the manual group increased to 251 s (201 vs 251, p < 0.05). Participants' questionnaire results show comparable psychological stress in both groups. Medical staff expressed that using a robot would reduce their psychological stress.

**Conclusions:**

We propose a safe and effective robotic technology for collecting nasopharyngeal specimens without face-to-face contact, which may reduce the stress of physicians and nurses. This technology can also be optimized for efficiency, making it useful in situations where droplet-transmitted infectious diseases are a concern.

## 1 Introduction

The coronavirus disease 2019 (COVID-19) pandemic has posed a significant threat to global public health. The primary mode of transmission of the coronavirus (SARS-CoV-2) is through person-to-person spread via respiratory droplets or aerosols [1]. Physicians and nurses are at a high risk of infection when performing aerosol-generating procedures, such as nasal or oral swabbing, and even when wearing proper personal protective equipment (PPE) [2].

Nasopharyngeal swabs are the gold standard for diagnostic testing of SARS-CoV-2 [3]. To reduce the risk of cross-infection between medical staff and patients during the collection of nasopharyngeal specimens, various protective measures have been implemented, such as outdoor temporary medical stations, plastic face shields, and acrylic sampling windows [4]. However, these measures can still present challenges in protecting both healthcare providers and patients. A "zero-contact" approach might provide a solution to prevent cross-infection between healthcare staff and patients, as well as alleviate the physical and psychological pressure faced by medical staff during the pandemic.

During the COVID-19 outbreak, there was a shortage of well-trained healthcare providers available to collect nasopharyngeal specimens, resulting in a decrease in the quality of the specimens and false-negative results [5,6]. The use of a robotic sampling system has the potential to address this issue by standardizing the specimen collection process. However, it is not yet ready for clinical use [[7], [8], [9], [10], [11], [12], [13]].

The purpose of this study is to determine if the nasopharyngeal swab robot (NSR) can effectively and safely collect nasopharyngeal specimens, as well as to assess whether it could reduce the psychological stress of physicians and nurses during potential future pandemics caused by droplet-transmitted infectious diseases.

## 2 Methods

### 2.1 Study design and setting

This prospective controlled study was conducted in two teaching hospitals in Taiwan, with an estimated annual number of emergency visits of 60,000 and 40,000 patients, respectively. Eighty adult subjects were included in the study; 40 of these subjects had their nasopharyngeal specimens collected by the inspection system robot, constituting the experimental group, while the remaining 40 subjects had specimens collected by medical staff using the manual method, which was considered the control group. The inclusion criteria were as follows: (1) participants aged over 20; (2) subjects who visited the emergency department and were categorized at level five on the 5-level triage scale; (3) subjects who requested SARS-CoV-2 testing due to various reasons such as job requirements, infection concerns, or travel-related departure needs; (4) subjects who were ambulatory and could follow verbal commands. The exclusion criteria were: (1) apparent deformity of the nose structure; (2) known nasopharyngeal neoplasm; (3) known coagulopathy; and (4) history of nasal surgery. Participants were provided with a detailed explanation of the study's procedures, risks, and benefits, and they provided written informed consent prior to participating. After watching a 1-min video on the system robot's collection process, subjects could choose whether to accept collection by the system robot, according to their personal preferences. Those who did not want the collection to be carried out by the robot were arranged to have their specimens collected by medical staff. The time spent on specimen collection, wearing PPE, and any adverse events were recorded during collection. The technician operating the robot and the participant were in two separate, isolated spaces and so they did not need to wear PPE. However, the medical staff in the manual sampling group needed to record the time spent wearing PPE. After the collection was completed, subjects and medical staff were asked to fill out a feedback questionnaire. This study was granted ethical approval by the Institutional Review Board (IRB) of China Medical University Hospital in Taiwan (IRB number: CMUH109-REC1-105). The IRB follows Good Clinical Practice guidelines and adheres to relevant laws and regulations in its organization and operation.

### 2.2 Real-time reverse transcription-polymerase chain reaction (RT-PCR) for SARS-CoV-2 RNA detection

We utilized the LightMix® 2019-nCoV real-time RT-PCR assay (Tib-Molbiol, Germany, licensed from Roche Diagnostics) [14] to detect the E and RdRp regions of SARS-CoV-2. If these regions tested positive, the presence of SARS-CoV-2 was then confirmed by detecting the N gene. RT-PCR was performed using a Cobas z 480 Analyzer (Roche), with thermal cycling conditions set according to the manufacturer's instructions.

Human ribonuclease P (RP) served as an internal control to ensure sample quality. The human RP-specific probe was labeled with Cy5 dye, allowing the detection of the amplified products. Samples were considered positive if the fluorescence curves crossed the cycle threshold (Ct) within 40 cycles, in accordance with the recommendations of the United States Centers for Disease Control and Prevention (CDC) [15]. To enhance the precision of the test, the threshold should be situated within the exponential phase of the fluorescence curve, above any background noise. Consequently, to achieve increased precision and stricter control, our hospital adopted a threshold of 35 cycles (≤35 Ct). If the RP fails to amplify (Ct > 35), it could indicate inadequate nucleic acid extraction, insufficient human cells, improper assay setup and execution, or reagent malfunction. Such samples were marked as 'invalid,' and a new nucleic acid extraction was performed.

### 2.3 Outcome measures

This trial evaluated two primary outcomes: specimen quality and success rate of nasopharyngeal swab collection. The specimen was considered valid if its Ct value was less than or equal to 35 (≤35 Ct). If the Ct value was greater than 35, the specimen was classified as unqualified.

In our study, an attempt at collection was considered successful if it was completed on the first attempt. If more than one attempt was needed, due to bending of the soft sampling rod or failure to insert it into the nasopharynx, it was considered a failure. However, only one swab sample per subject was sent to the laboratory for testing, regardless of the number of attempts.

The secondary outcomes of the trial included pain index during the nasopharyngeal collection process, psychological stress of the subjects and medical staff, and the time spent collecting the nasopharyngeal specimen. Pain was rated on a scale of 0–10, with 0 indicating "no pain" and 10 being "very much pain”. After the nasopharyngeal collection, a questionnaire survey was conducted among the subjects and medical staff. The survey responses were scored on a five-point Likert scale, with responses of “Strongly Agree”, "Agree,” "Neutral,” "Disagree,” and "Strongly Disagree" being given scores of 1–5 points, respectively.

The inspection time via NSR was calculated after the testing subject was placed in the inspection position. The process included the time taken to fix the head on the chin holder, the time for the nasopharynx facial recognition software to customize the robotic arm, the time to position the robotic arm, and the time for the nasopharyngeal swab to enter the nasal cavity for collection. The manual sampling time included the time to put on PPE, the time to position the patient in the sampling position, and the time for the nasopharyngeal swab to enter the nasal cavity for specimen collection.

### 2.4 Robotic technology

**Fig. 1:**
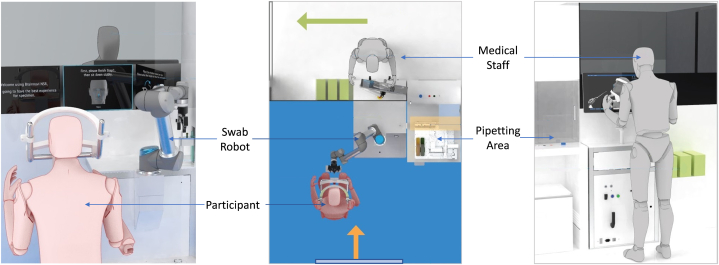
Setup for Specimen Collection Room. The participant and healthcare staff were situated in separate cabins, separated by a transparent shield to prevent cross-infection. An integrated microphone system ensured seamless communication.

**Fig. 2:**
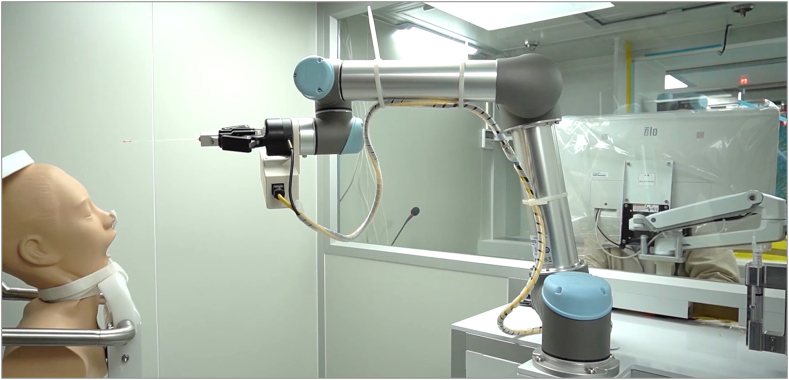
Setup for Robotic Specimen Collection. A chin holder stabilized the participant's head in the sniffing position, facilitating specimen collection by the robotic arm. This robotic arm is equipped with six multi-directional joints, a finger gripper, and a facial scanner.

The NSR consists of a compact robotic arm and display screen mounted on a wheeled trolley. Different types of nasopharyngeal swabs can be attached to the end of the arm (resembling a finger gripper), according to the requirements of the procedure. The patients and healthcare staff were placed in separate cabins with a transparent shield between them to prevent the risk of cross-infection. (Fig. 1). The chin holder was used to stabilize the patient's head and was designed to be open so that the patient could withdraw at any time if they felt uncomfortable (Fig. 2). Verbal commands from the technician were used to ease anxiety in participants. Additionally, a confirmation prompt appeared on the monitor before the robotic arm began moving, and an immediate termination feature was activated when the technician released the foot-controlled pedals.

**Fig. 3:**
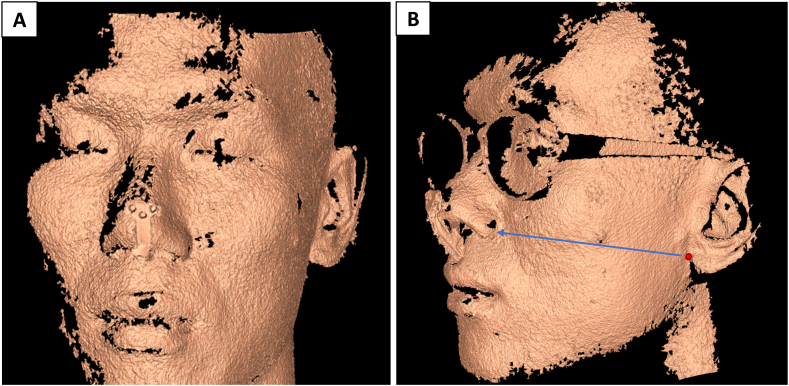
Facial Recognition for Nasopharyngeal Depth Estimation. (A) Facial recognition technology reveals the three-dimensional structure of a participant's face. (B) The distance measured between a participant's nostrils and ears is used to determine nasopharyngeal depth.

**Fig. 4:**
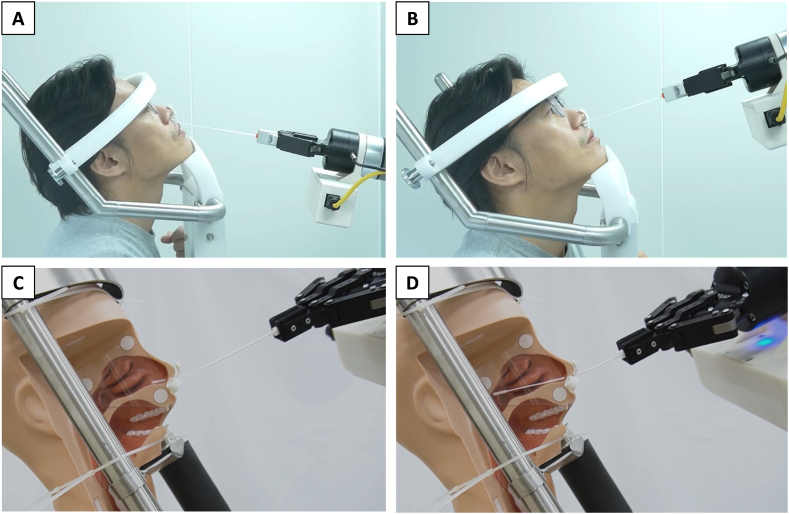
Nasopharyngeal Specimen Collection Steps Using the Swab Robot. (A) The robot inserts the swab 1 cm into the nasal vestibule. (B - C) The swab is elevated to a 30-degree angle, aligning it parallel to the floor of the nasal fossa. (D) The swab is gently rotated and advanced straight to the posterior wall of the nasopharynx.

The NSR has received Conformité Européenne (CE) certification as a Class I medical device and has been granted Emergency Use Authorization (EUA) by the Taiwan Food and Drug Administration (FDA) for use during the COVID-19 pandemic. The NSR is equipped with facial recognition technology that allows it to accurately measure the distance between a patient's nostrils and ears, which helps determine the depth of the nasopharynx [Fig. 3(A and B)]. Utilizing a flexible swab (brand name: FLOQSwabs®, Copan Diagnostics Inc., USA), the robot first inserts the swab 1 cm into the nose vestibule, and then elevates it at a 30-degree angle so it aligns parallel to the floor of the nasal fossa, before proceeding straight to the posterior wall of the nasopharynx [16] [Fig. 4(A -D) and supplemental video]. With both clockwise and counterclockwise rotations, the robot repeatedly rubs the swab against the posterior wall of the nasopharynx for 10 s, retracts it, and immerses it into the transport medium (UTM, Universal Transport Medium®) without additional stirring or shaking, thus completing the sampling process. The robot is easy to use and has a user-friendly interface that allows healthcare staff to quickly learn how to safely perform the tests.

Supplementary data related to this article can be found online at doi:10.1016/j.heliyon.2023.e20757

The following are the Supplementary data related to this article:

Video 1 Video 1

### 2.5 Statistical analysis

The statistical analysis software SPSS 13.0 for Windows was used to analyze the data. The demographic information, swab quality, and pain scale of the participants were described using statistical measures, such as means and standard deviations for continuous data, and numbers and percentages for categorical data. To compare the robotic and manual groups, t-tests were used for continuous variables and chi-square or Fisher's exact tests were used for categorical variables. Statistical *p* values of less than 0.05 indicated statistical significance.

## 3 Results

### 3.1 Patient profile and baseline demographics

During the study period from September 23 to October 27, 2020, a total of 80 research subjects were enrolled. All the subjects were ambulatory and visited the emergency department for COVID-19 sampling. Table 1 provides a summary of the demographic characteristics and the results of the univariate analysis for robotic and manual sampling among the enrolled patients. Of the 80 testing subjects, 9 were foreigners, originating from countries including Korea, India, Egypt, Vietnam, Italy, and Macao. The subjects' ages ranged from 22 to 75 years old, with 9 subjects being over the age of 60 and 71 subjects under 60 years old. The majority of the subjects were male, with 87.5 % in the nasal robot group (experimental group) and 77.5 % in the manual collecting group (control group). There was a high level of homogeneity between the two groups.

**Table 1:** Demographic data, swab quality, and pain scale of participants with robotic and manual sampling.

Variables	Robotic group (n = 40)	Manual group (n = 40)	
	Mean ± SD/N(%)	Mean ± SD/N(%)	*p*-value
Age (years old)	42.4 ± 11.8	46.6 ± 13.3	0.15
20-30	8	6	
31-40	11	8	
41-50	12	10	
51-60	6	10	
>60	3	6	
Gender			0.33
Male	35 (87.5)	31 (77.5)	
Female	5 (12.5)	9 (22.5)	
Cycle Threshold (Ct)			
Hospital 1	30.1 ± 1.8	27.6 ± 2.0	<0.01
Hospital 2	31.6 ± 1.3	28.3 ± 1.6	<0.01
Overall	30.9 ± 1.7	28.0 ± 1.8	<0.01
*Sampling Attempt*			
Success on the First Attempt	37 (92.5)	40 (100)	
Needing a Second Attempt	3 (7.5)	0 (0)	
Pain Scale (0–10)	2.8 ± 1.7	3.6 ± 2.0	0.07
Sampling Time (seconds)			
Swabbing	201 ± 37.2	29 ± 5.5	<0.01
Swabbing plus wearing PPE		251 ± 36.1	<0.01

Abbreviations: SD, standard deviation; PPE, personal protective equipment.

### 3.2 Specimen quality and success rate of nasopharyngeal swabbing in first attempt

The average Ct value in the robotic group was 30.9, whereas that in the control group was 28.0. All samples had Ct values of <35, indicating that they were of good quality in both groups. However, when comparing the Ct values, there was a statistically significant difference between the two groups.

In each group, 40 subjects completed the collection of NP specimens, and no participants dropped out during the process. Only one swab sample per subject was sent to the laboratory, regardless of the number of attempts. However, in the robotic group, three of the 40 subjects required a second attempt to complete the specimen collection, resulting in a success rate of 92.5 % (37/40).

### 3.3 Pain scale and time for specimen collection

The average pain index was 2.8 in the robotic group and 3.6 in the control group. Although there was no statistically significant difference between the two groups, using the NSR for nasopharyngeal specimen collection was perceived as more comfortable.

The robotic group had an average sampling time of 201 s, while the manual group had an average sampling time of 29 s. However, when considering the time needed to put the PPE on, the average sampling time for the manual group increased to 251 s, which was significantly longer than that of the robotic group. The technician operating the robot could do so remotely from a separate cabin and did not need to wear PPE, making the robotic group more time-efficient.

### 3.4 Questionnaire for subjects

Eighty participants completed a psychological stress assessment questionnaire for nasopharyngeal examination. The results for the five assessment questions are presented in Table 2. The percentage of participants who did not feel anxious during the procedure was similar in both the robotic and manual groups (50 % vs. 57.5 %, p = 0.76). In the robotic group, 65 % of participants were not concerned about potential infection even if they knew the previous participant tested positive for COVID-19, although this difference was not statistically significant (p = 0.07). Only 12.5 % of participants expressed regret about the method they chose, with no significant differences being observed between the robotic and manual groups (12.5 % vs. 12.5 %, p = 1.00). A significantly higher percentage of subjects in the robotic group (70 %, p < 0.05) found that nasopharyngeal specimen collection was less uncomfortable than they had imagined, compared with 45 % in the manual group. The results of the last question showed that both methods were accepted by the participants and that they were willing to recommend them to others (77.5 % vs. 77.5 %, p = 1.00).

**Table 2:** Questionnaire for subjects - psychological response to different nasopharyngeal specimen collection methods.

	Robotic group, n = 40 (%)					Manual group, n = 40 (%)					
	SA	A	N	D	SD	SA	A	N	D	SD	*p*
**1. Do you feel anxious during the sampling process?**	2(5)	5(12.5)	13(32.5)	1(2.5)*	19(47.5)*	3(7.5)	3(7.5)	11(27.5)	11(27.5)*	12(30)*	0.76
**2. Does knowing the method may have been used on an infected person worry you?**	0(0)	6(15)	8(20)	8(20)*	18(45)*	1(2.5)	12(30)	7(17.5)	10(25)*	10(25)*	0.07
**3. Do you regret choosing the above sampling method?**	1(2.5)*	4(10)*	8(20)	10(25)	17(42.5)	0(0)*	5(12.5)*	11(27.5)	17(42.5)	7(17.5)	1.00
**4. Is the method you chose less uncomfortable than you imagined?**	14(35)*	14(35)*	8(20)	2(5)	2(5)	4(10)*	14(35)*	10(25)	6(15)	6(15)	0.03
**5. Would you recommend this method to others?**	17(42.5)*	14(35)*	8(20)	1(2.5)	0(0)	6(15)*	25(62.5)*	6(15)	1(2.5)	2(5)	1.00

In each question, the categorical data with the symbol “*” are summed for Pearson chi-square test.

Abbreviation: SA, strongly agree; A, agree; N, neutral; D, disagree; SD, strongly disagree.

### 3.5 Questionnaire for medical staff

Fourteen healthcare providers participated in the study, with seven choosing to use the robot for nasopharyngeal specimen collection and seven opting for manual collection. As shown in Table 3, the statistical analysis included assessments of psychological stress and behavioral impact. Physicians and nurses generally prefer using robots for nasopharyngeal specimen collection because of the lower level of psychological stress compared with the manual method. When using the robotic method, they felt less stressed about completing the procedure on time, had fewer concerns about becoming infected, and were less worried about wasting excessive PPE. In addition, the decision to use the robotic method was associated with lower levels of overall anxiety. During the COVID-19 pandemic, physicians and nurses have adjusted their behaviors regarding safety procedures. They have tended to modify their operating procedures during nasopharyngeal sampling, to wear more protective equipment beyond the standard PPE, and to live in hospital accommodations to avoid potentially infecting their families. Both the robotic and manual groups displayed a strong preference for technological assistance, with all participants (14 out of 14) strongly agreeing or agreeing with the idea of using a sampling robot to assist in nasopharyngeal specimen collection.

**Table 3:** Questionnaire for physicians and nurses- psychological stress and behavior impact assessment.

		Robotic Group, n = 7					Manual Group, n = 7					
		SA	A	N	D	SD	SA	A	N	D	SD	
Psychological stress assessment												
	**1. Do you feel stressed about completing the procedure on time when using the above method?**	1	1	1	1	3	3	3	0	0	1	
	**2. Are you concerned about becoming infected when deciding to use the above method?**	0	0	1	0	6	3	4	0	0	0	
	**3. Do you worry about wasting too much PPE with the above method?**	0	0	0	2	5	3	3	1	0	0	
	**4. Does deciding to use the above method make you anxious?**	0	0	0	2	5	0	4	2	1	0	
**Behavior impact assessment**												
	**1. Would you modify your specimen collection procedure to prevent infection?**	3	3	0	0	1	1	3	0	0	3	
	**2. Would you wear extra protective equipment in addition to the standard PPE?**	3	4	0	0	0	4	3	0	0	0	
	**3. Would you consider living in hospital accommodations to avoid infecting your family?**	2	3	0	2	0	4	1	2	0	0	
	**4. Would you prefer using a sampling robot to assist with the collection of nasopharyngeal specimens?**	7	0	0	0	0	5	2	0	0	0	

Abbreviation: SA, strongly agree; A, agree; N, neutral; D, disagree; SD, strongly disagree; PPE, personal protective equipment.

### 3.6 Data acquisition

There were no missing data, adverse events, or subject withdrawals during the study period.

## 4 Discussion

To the best of our knowledge, this is the first controlled study to demonstrate the use of a novel nasal swab robot in real patients during the COVID-19 pandemic. Our study showed that the use of a robot to collect respiratory specimens is a safe, effective, and useful method compared to manual sampling. However, our study also identified some defects in the nasal swab robot that should be addressed in the future.

The United States CDC generally recommends Ct values < 40 as an indicator of SARS-CoV-2 RNA positivity [15,17]. However, variations in reagents, protocols, and analysis methods for RT-PCR assays have led to different Ct values being used for COVID-19 diagnosis across various regions [18]. Therefore, it is worth noting that the positive and negative Ct cutoff values should be locally validated. In alignment with our study outcomes, our laboratory adopted protocols from the US CDC and referred to several publications in the literature [14,15]. choosing a Ct cut-off value of 35. This value complies with the regulations of the Ministry of Health and Welfare of Taiwan, and represents a more stringent method for assessing sample quality. Specifically, failure to amplify the human RP gene within 35 cycles generally indicates unqualified specimens, often resulting from inadequate human cells. By selecting a cutoff value of 35, we aimed to ensure a higher standard of specimen quality, enhancing the reliability and accuracy of our testing.

When comparing specimen quality between the robotic and manual groups, although a statistical difference was observed, it did not affect clinical practice. The parameter settings of the robotic arm may be a reason for these differences. The robotic arm precisely reached the nasopharynx with a highly accurate nasal cavity pathway and then gently rotated the nasopharyngeal swabs, resulting in a qualified specimen with relatively low pain. In contrast, manual sampling often requires adjusting the angle of entry into the nasopharynx and rubbing the turbinate mucosa several times, which may produce obvious discomfort, despite a low Ct value. In addition, the robotic arm gently immerses nasopharyngeal swabs in the liquid transport medium, which may result in fewer specimens falling into the liquid. This can be improved using external shaking or additional stirring to reduce the interference of this factor (as shown in the supplemental video). One study [9] found that using a mechanical arm with a larger rotational force for oropharyngeal sampling caused the test subjects to feel more pain. This finding highlights the need to balance optimal specimen quality with patient comfort and safety.

In the robotic group, three of the 40 subjects required a second attempt to achieve the sampling process. Given that everyone's nasal structures are different, the flexible sampling rod will bend when encountering resistance, making it unable to pass through smoothly. If pressure sensors, soft grippers, and soft wrist designs are installed on a robotic arm [7,13], making the movements more precise, this problem may be solved. Photographic techniques can be used to create a 3D facial structure; however, this is an external rather than an internal structure. Some studies have used an endoscopic system or real-time images to solve this problem [8,9]; however, this would require a higher learning curve and trained personnel. Whether this method is adopted must be carefully weighed against its advantages and disadvantages.

Previous research has shown that the COVID-19 pandemic can exacerbate the condition of patients with pre-existing mental disorders [19] and even cause psychological distress in patients who have not previously experienced mental disorders [20]. Therefore, it is important to minimize the stress experienced by the subjects during nasal and throat swab testing. The results of our questionnaire revealed similar levels of psychological stress among participants who chose robotic and manual sampling. However, the potential to enhance patient experience through technological innovation remains. Natural language chatbots can provide empathetic interactions, thus easing patient anxiety [21]. The integration of force sensors into the robotic arms can minimize discomfort by avoiding over irritation of the nasal cavity [7]. Moreover, implementing a system that can scan facial structures and rapidly tailor the depth and route could reduce the time that patients spend in the sampling room, thereby alleviating stress [22]. Further multidisciplinary cooperation is required.

In addition, from the questionnaire responses of physicians and nurses, we understood that the medical staff had a heavy workload during the pandemic. The need to complete testing tasks within a certain timeframe while avoiding infection has caused significant psychological stress in physicians and nurses. As a result, physicians and nurses have made some modifications to the nasal and throat swabbing process, such as requiring testing subjects to turn sideways or stand behind an acrylic window [4] to prevent direct cross-infection, and wearing a surgical mask on top of an N95 mask. Under this pressure, physicians and nurses have consistently believed that the use of a testing robot would significantly reduce psychological and work-related stress.

If we do not consider the time it takes to wear the PPE, manual sampling requires an average of only 29 s, which is much lower than the time it takes for a robot to sample. Therefore, for large-scale outdoor screening, manual sampling is more effective for medical personnel. Robotic sampling has several general advantages however. First, the procedure is performed by a single technician, which saves medical personnel and reduces psychological stress. Second, it standardizes the sampling process, produces a lower pain scale score, and is as safe and effective as the manual sampling methods. Third, it is available 24 h a day and can be remotely controlled to avoid face-to-face contact. In the future, if a robotic system can reduce its sampling time and become fully automated without the need for a technician to operate it, its usability will greatly improve.

### 4.1 Limitations

First, this study has a selection bias because the robotic device was designed for individuals who can follow instructions. Consequently, the trials did not include uncooperative or resistant subjects, which may limit the randomness and double-blind nature of the study. Additionally, individuals with a fear of undergoing robotic sampling, known nasopharyngeal tumors, a history of nasal surgery, or known coagulopathy were excluded from the study.

Secondly, pain perception is highly subjective. The discomfort of patients could have been better assessed if the same individuals were able to compare the robotic and manual methods. However, given the constraints during the peak of the COVID-19 pandemic and the potential implications for subjects' rights, such as quarantine requirements or travel restrictions, this approach was not feasible in our study.

Third, the sample size (80 participants) was small. Our study was conducted at the peak of the COVID-19 pandemic, which made it challenging to execute a clinical trial with a larger database. Moreover, because the robotic sampling technique is novel and not yet fully developed, we conducted a preliminary study to evaluate its feasibility and effectiveness. Further large-scale trials are required to validate these findings.

## 5 Conclusions

In this preliminary study, which involved a limited number of patients, we introduced a robotic technology for collecting nasopharyngeal specimens and demonstrated its safety and effectiveness. This method minimizes face-to-face contact and alleviates psychological stress experienced by physicians and nurses. With further optimization of the time efficiency, this robotic approach could be useful in situations where infectious diseases transmitted by droplets are a concern.

## Data availability statement

The data is included in both the manuscript and the supplemental material of our article.

## CRediT authorship contribution statement

**Jiun-Hao Yu:** Conceptualization, Data curation, Formal analysis, Investigation, Methodology, Project administration, Resources, Supervision, Validation, Writing – original draft, Writing – review & editing. **Sung-huai Hsieh:** Methodology, Software, Validation, Writing – review & editing. **Chieh‐Hsiao Chen:** Conceptualization, Resources, Software. **Wen-Kuan Huang:** Supervision, Writing – review & editing.

## Funding statement

No funding was received for this study.

## Declaration of competing interest

All authors have no financial interests to declare. Jiun–Hao Yu reports equipment was provided by Brain Navi Biotechnology Co., Ltd., Hsinchu, Taiwan. Co-author Chieh‐Hsiao Chen was employed by Brain Navi Biotechnology Co., Ltd., the company responsible for providing robotic arms and technical support for our project. It's important to clarify, however, that no financial support was received from the company. Instead, all the clinical and laboratory data crucial to our work were exclusively managed and overseen within an academic institution.
