# Supplementary material for: Comparison of the safety, effectiveness, and usability of swab robot vs. manual nasopharyngeal specimen collection
Source: Heliyon. 2023 Oct 13;9(10):e20757. doi: 10.1016/j.heliyon.2023.e20757 (PMC10597818; doi:10.1016/j.heliyon.2023.e20757)
Supplement: Multimedia component 1 [file mmc1.doc]

**Questionnaire for Conducting Nasal Swabs for COVID-19 Testing**

**Date: Year Month Day Signature:**

**Ⅰ. General Information**

| **1.Certificated level of emergency responsibility hospital：**  □Advanced □Intermediate □Basic □Non-responsibility hospital  **2.Gender：**□Male　□Female  **3.Age(in years)：**□<20 □21-30 □31-40 □41-50 □51-60 □>61  **4.Job Title：**□Resident □Attending Physician □Nurse Practitioner  **Year in business：** **Department:** □Emergency Medicine □  **5.How many people received COVID-19 testing per day in your workplace：**  □<5 □6-10 □11-15 □16-20 □21-25 □  **6.Is a specialist put in charge of the COVID-19 testing in your workplace：**  □Yes □No |
| --- |

**II. Psychological Response of Subjects to Different Collection Methods**

| **SA**:Strongly Agree; **A**:Agree; **N**:Neutral; **D**:Disagree; **SD**:Strongly Disagree | | | | | | |
| --- | --- | --- | --- | --- | --- | --- |
|  | **Nasal swabbing is conducted by：**  □**Healthcare provider** □**Robot** | **5**  **SA** | **4**  **A** | **3**  **N** | **2**  **D** | **1**  **SD** |
| 1. | Do you feel anxious during the sampling process? | □ | □ | □ | □ | □ |
| 2. | Does knowing the method may have been used on an infected person worry you? | □ | □ | □ | □ | □ |
| 3. | Do you regret choosing the above sampling method? | □ | □ | □ | □ | □ |
| 4. | Is the method you chose less uncomfortable than you imagined? | □ | □ | □ | □ | □ |
| 5. | Would you recommend this method to others? | □ | □ | □ | □ | □ |

**III. Impact of COVID-19 on Psychological Stress and Behavior of Healthcare Providers**

| **SA**:Strongly Agree; **A**:Agree; **N**:Neutral; **D**:Disagree; **SD**:Strongly Disagree | | | | | | |
| --- | --- | --- | --- | --- | --- | --- |
|  | **Nasal swabbing is conducted by：**  □**Healthcare provider** □**Robot** | **5**  **SA** | **4**  **A** | **3**  **N** | **2**  **D** | **1**  **SD** |
| 1. | Do you feel stressed about completing the procedure on time when using the above method? | □ | □ | □ | □ | □ |
| 2. | Are you concerned about becoming infected when deciding to use the above method? | □ | □ | □ | □ | □ |
| 3. | Do you worry about wasting too much personal protective equipment with the above method? | □ | □ | □ | □ | □ |
| 4. | Does deciding to use the above method make you anxious? | □ | □ | □ | □ | □ |
| 5. | Would you modify your specimen collection procedure to prevent infection? | □ | □ | □ | □ | □ |
| 6. | Would you wear extra protective equipment in addition to the standard personal protective equipment? | □ | □ | □ | □ | □ |
| 7. | Would you consider living in hospital accommodations to avoid infecting your family? | □ | □ | □ | □ | □ |
| 8. | Would you prefer using a sampling robot to assist with the collection of nasopharyngeal specimens? | □ | □ | □ | □ | □ |

Abbreviation: COVID-19, coronavirus disease 2019
